# Editorial: Food of the future: insects

**DOI:** 10.3389/fnut.2023.1269008

**Published:** 2023-10-10

**Authors:** Benjamin Kumah Mintah, Mokhtar Dabbour

**Affiliations:** ^1^CSIR–Food Research Institute, Accra, Ghana; ^2^Department of Agro-Processing Technology and Food Bio-Sciences, CSIR College of Science and Technology (CCST), Accra, Ghana; ^3^Department of Agricultural and Biosystems Engineering, Faculty of Agriculture, Benha University, Qalyubia, Egypt

**Keywords:** insects farming, bioactivity, novel technology, protein, entomophagy, ultrasound

Interest in alternative food sources to address the challenges of feeding a growing global population while taking into account sustainability and resource use has increased lately. One such category of alternative food source (food of the future) is edible insects, which (globally) are consumed by many cultures. They are not only considered sustainable, but also nutritious food source (1–3), due to their rich protein, vitamin (including B vitamins), healthy fat, and essential mineral (e.g., zinc—Zn, and iron - Fe) contents. Insects have, as well, several other benefits to humans and the environment. Compared to conventional livestock, the raising and/or breeding of insects requires significantly fewer or less resources (e.g., water, and land) (4). They latently and efficiently convert feed (organic/kitchen waste) into edible and nutritious body mass (5), contributing to reducing environmental waste.

The acceptance of insects as food source (by consumers worldwide) is also gradually increasing, driven by their nutritional, health and environmental benefits, as well as gastronomic advances. However, cultural and individual perceptions linked to the association of insects to waste/pests still pose a challenge to their complete adoption, not overlooking the flip-feeling of disgust. Consequently, researchers are channeling their efforts into improving the efficiency and hygiene of insect farming, isolating components like protein, developing and optimizing insect-based food/functional food products, and addressing potential risk associated with allergens and other contaminants. It follows that, the widespread acceptance of insects as food that has the potential to diversify global food sources in few years to come, will not only depend on factors such as food processing technologies, advances in farming, individual or cultural perceptions, but also education/training, and regulatory framework.

This Research Topic, therefore, focused on multiple subjects on insects as future food source, with collection of articles on safety, processing, and novel technologies and strategies toward increasing their application and consumption. The articles discuss the digestibility and quality of insect protein, willingness to consume edible insects, refining the biomass of some insects into valuable fractions using novel and conventional methods, and feed for insect farming.

Considering the importance of protein quality measurement, as in helping to know how well proteins would meet the physiological needs of humans, Hammer et al. used an *in vitro* digestion technique based on indispensable amino acid scores to assess the quality of *Acheta domesticus* and *Tenebrio molitor* protein, while considering the influence of food preparation and processing as well. Compared to chicken breast, their observation pointed to it that, by using appropriate food preparation/processing method, the proteins from *A. domesticus* and *T. molitor* could be suitable alternative to that from conventional sources (specifically animals) and would especially benefit children (≥6 months).

Hopkins et al., on food neophobia, examined the relation with dietary choices and consumer readiness to adopt novel foods like insects; with the aim of increasing the consumption of insects in the near future or as food of the future. Part of their report showed strong correlation between food neophobia and respondents' dietary choices (vegetarian or vegan diet); and decreased inclination to the consumption of insects in the future. Lower food neophobia were also reported for respondents who eat insects than those who do not - non-insect eaters (6). Considering that food neophobia forms eating habits for further life, right from early childhood (7), it is logical to recommend the introduction of entomophagy at age ≤8 years, where a child is sensitive to the immediate environment. Likewise, for other groups/individuals aligned with aversive entomophagy, particularly driven by dislike of unfamiliar food (like insects), a recommendation through the promotion of tasting sessions could be vital to reducing food neophobia and increase the adoption of insects as food.

Currently, researchers are applying novel technologies to aid the extraction and as well alter the functionality of targeted isolates such as protein (8). While these novel technologies have been found to enhance the extractability and functional attribute of isolates of interest (9), there are also instances that show conflicting reports (5). Psarianos et al., among other objectives, focused on separating the component of house crickets into one/many products (fat, phenolics, proteins and chitin), using novel or emerging technology. They showed how ultrasound and high pressure processing could affect, for example, the phenolic yield and antioxidant activity differently. The use of eutectic solvent and urea in the sepearation of chitin and protein, and how such impacted the protein content was also explored by the authors.

While finding ways to increase the adoption of insects as food source, the sustenance of insects through Small-scale farming is imperative. Toward finding low-cost, but reliable, feeds for insects, Ventura et al. investigated the use of maize crop residue to produce a feed adjunct (that may be economical) for the farming of cricket (*Gryllus bimaculatus*), making use of mushroom (*Pleurotus ostreatus*) mycelium. An aspect of their results pointed to high bioavailable iron in the insect. Also on bioactivity, Torres-Castillo et al., reported on the role of insects as source of antioxidant and/or phenolic compounds for industrial applications.

In sum, the collection of articles (in this Research Topic) highlights some novel examples (technologies and/or strategies) that could contribute to the adoption of insects as food source in the next few decades. Granting that research on entomophagy is still at its early stage, we are of the view that these latent and manifestly beneficial arthropods qualify to be one of the main future food source that could help meet the nutritional needs of humans, and also conservation of the planet.

## Author contributions

BKM: Writing—original draft, Writing—review and editing. MD: Writing—review and editing.
